# How to limit the burden of data collection for Quality Indicators based on medical records? The COMPAQH experience

**DOI:** 10.1186/1472-6963-8-215

**Published:** 2008-10-21

**Authors:** Clément Corriol, Valentin Daucourt, Catherine Grenier, Etienne Minvielle

**Affiliations:** 1 CERMES – INSERM U750 – COMPAQH, Hôpital de Bicêtre, Le Kremlin-Bicêtre, France; 2 REQUA (Quality Network of Hospitals in Franche-Comté), Besançon, France; 3 FNCLCC (French Federation of Cancer Centres), Paris, France

## Abstract

**Background:**

Our objective was to limit the burden of data collection for Quality Indicators (QIs) based on medical records.

**Methods:**

The study was supervised by the COMPAQH project. Four QIs based on medical records were tested: medical record conformity; traceability of pain assessment; screening for nutritional disorders; time elapsed before sending copy of discharge letter to the general practitioner. Data were collected by 6 Clinical Research Assistants (CRAs) in a panel of 36 volunteer hospitals and analyzed by COMPAQH. To limit the burden of data collection, we used the same sample of medical records for all 4 QIs, limited sample size to 80 medical records, and built a composite score of only 10 items to assess medical record completeness. We assessed QI feasibility by completing a grid of 19 potential problems and evaluating time spent. We assessed reliability (κ coefficient) as well as internal consistency (Cronbach α coefficient) in an inter-observer study, and discriminatory power by analysing QI variability among hospitals.

**Results:**

Overall, 23 115 data items were collected for the 4 QIs and analyzed. The average time spent on data collection was 8.5 days per hospital. The most common feasibility problem was misunderstanding of the item by hospital staff. QI reliability was good (κ: 0.59–0.97 according to QI). The hospitals differed widely in their ability to meet the quality criteria (mean value: 19–85%).

**Conclusion:**

These 4 QIs based on medical records can be used to compare the quality of record keeping among hospitals while limiting the burden of data collection, and can therefore be used for benchmarking purposes. The French National Health Directorate has included them in the new 2009 version of the accreditation procedure for healthcare organizations.

## Background

Medical records are a key instrument in the coordination of patient care. They facilitate diagnosis and information sharing, reduce medical errors, and serve an important medical-legal function [1], regardless of the type of healthcare organisation (HCO) – public or private – or clinical specialty. However, when the first accreditation results for French HCOs were made public in 2003 their quality was found to be in need of substantial improvement. Two frequent shortcomings were no written record of the information provided to patients and unsigned drug prescriptions [2]. Ever since the French health authorities announced that each person with national health insurance coverage will have a single electronic medical record shared between patient and health practitioners, they have become a hot topic [3].

Medical records are also used as a source of clinical data for documenting Quality Indicators (QIs) [4-6], although other methods providing more relevant data, such as standardized patients or vignettes [7-9], are also available. However, since the latter have not been used on a wide scale to compare hospitals, collecting data for QIs from medical records remains the standard despite the workload and expense of chart abstraction. The estimated total cost of copying and reviewing 8 000 charts manually is US$10 millions [10].

Our study focuses on the need to improve the quality of medical records because of their key role in the coordination of care. We developed and tested a set of four QIs for medical records (record conformity, traceability of pain assessment, screening for nutritional disorders, and time elapsed before sending discharge letters) and collected data in a panel of 36 volunteer hospitals. We wanted to establish whether these QIs could be used by all types of hospital while limiting the burden of data collection. We determined QI feasibility, reliability, and ability to discriminate among the hospitals.

## Methods

The study was run by the COMPAQH project, a French national initiative for the development of QIs, coordinated by the French Institute for Health and Medical Research (INSERM), and sponsored by the Ministry of Health and National Health Directorate [11]. The project's overall objectives are to select and test QIs in order to monitor quality and performance in French hospitals, and design ranking methods and Paying-for-Quality programs.

### QI selection

In 2003, the French Ministry of Health and the French National Authority for Health (HAS), listed 8 priority areas in need of quality improvement: continuity of care, pain management, management of patients with nutritional disorders, iatrogenic risks, patient satisfaction, follow-up of practice guidelines, management of human resources, and access to care. A set of 42 QIs relating to these areas was established by COMPAQH. Four of these QIs were based on hospital medical records and corresponded to 3 priority areas: QI 1: conformity of medical records (continuity of care), QI 2: traceability of pain assessment (pain management), QI 3: screening for nutritional disorders (management of patients with nutritional disorders), and QI 4: time elapsed before sending a copy of discharge letters to general practitioners (continuity of care).

QI 1 describes the overall quality of the medical record and is given by a composite score based on 10 items relating to the contents of the record (item present or absent) (Table 1). The other three QIs are based on specific data within the medical records and are expressed as proportions.

**Table 1:** QI description

QI	Description
Medical record conformity	Composite score describing compliance with 10 items: presence of surgical report, delivery report, anaesthetic record, transfusion record, outpatient prescription, outpatient record, admission documents, care and medical conclusions at admission, drug prescriptions during stay, and overall organisation of record
Traceability of pain assessment	Proportion of records containing at least one pain assessment result (Number of records containing at least one result/N)
Screening for nutritional disorders	Proportion of records giving body weight at admission (Number of records giving weight at admission/N)
Time elapsed before sending discharge letters	Proportion of records containing a letter sent within 8 days (Number of records containing a letter/N)

N = total number of records audited

### Data collection

A panel of 36 volunteer hospitals (16 public, 7 private not-for-profits, and 13 private profit-making) took part in collecting data on the 4 selected QIs in 2005. They comprised 24 acute care establishments, 4 cancer clinics, 4 rehabilitation centres, and 4 psychiatric hospitals. The 4 QIs except for QI 2 (traceability of pain assessment) were applicable to all establishments; QI 2 applied to acute care hospitals only.

There were 6 steps to the data collection process: (1) Diffusion of an instructions brochure for each QI describing the protocol and items for which data was to be collected; (2) Nomination of a person within each hospital who would oversee data collection; (3) Random selection of 80 medical records; (4) Data collection by 6 CRAs who used each medical record to complete the quality assessment grid under the supervision of a physician; (5) Calculation of results; (6) Summary of the strengths and weaknesses of each QI, and diffusion of the validated instructions brochures to the bodies responsible for generalising QI use.

The burden of data collection was reduced by using: (i) a single sample of medical records for all 4 QIs. The person who oversaw data collection in each hospital randomly drew the records; (ii) a small sample size (80 medical records for 4 QIs) that was nevertheless large enough to reveal differences among hospitals [12,13]. However, because of exclusions, a second and even a third set of 30 records, was also drawn. The mean number of records abstracted in each hospital was 93 (range 80–132, median 86); (3) a limited number of items for the QI for medical record conformity. The initial list of 16 items was cut down to 10 items after a working group had identified feasibility problems relating to some items (see below).

None of the 4 QIs required adjustment.

### QI feasibility

We drew up a list of problems that the CRAs might encounter during data collection. A working group of 5 physicians and 5 hospital managers then examined whether the list was complete by checking it against a number of medical records and made observations on the wording used for each item in the list. Their comments were used to revise the instructions brochure prior to its testing by the CRAs. The final list comprised 29 pre-identified problems. The CRAs completed the 29-item form for each QI in each hospital and also recorded all non-listed problems they had to deal with (*e.g. *problems relating to clarity of instructions or to time spent in collecting data on a specific item). In each hospital, they also estimated the time taken to sample 80 medical records, retrieve the medical records from archives, record the data on paper, enter it on computer, and control its quality.

The working group validated the amended version of the instructions brochure which was then used to assess QI reliability and discriminatory power.

### QI reliability

In 6 hospitals, a sample of 20 out of the 80 medical records was coded by 2 different CRAs (double-blind data capture). Reliability was given by the Kappa (κ) coefficient. A κ value greater than 0.6 indicates satisfactory reliability; a value above 0.8 indicates very satisfactory reliability [14]. For QI 1 (medical record conformity), we also computed the Cronbach α coefficient to assess the score's internal consistency and calculated inter-item correlations to establish whether any items might not provide redundant information [15].

### Discriminatory power

The hospitals were ranked into 5 categories using the Ontario Hospital Report method [16]. We chose this method because of the need to introduce uncertainty (*i.e. *confidence intervals, CI) into the ranking as the sample of medical records supporting the data was small [17]. The discriminatory power of each QI was assessed by comparing the results for each hospital. The hospitals were ranked into 5 groups on the basis of the overall mean 90% and 99% confidence intervals (CI) as indicated in the footnote to Table 3.

## Results

### Burden of data collection

The estimated time taken to collect data on the 4 QIs in each hospital was 8.5 days, broken down as follows: 0.5 days to sample the medical records, 2 days to retrieve the medical records from archives, 4 days to abstract the sample, 1 day to enter the data on computer, and 1 day to check data quality.

### QI feasibility

The rates of occurrence of the 29 pre-identified feasibility problems are given in Table 2. Overall occurrence rate was 16.6% (730 items corresponding to an identified problem divided by a total of 4404 coded items). Misunderstanding the QI was the most common problem (range 38.5 – 44.9% of coded problems). Three items were never a feasibility problem. Two were related to external events (i.e. low implication/motivation due to external events unrelated to the project, after data collection, and staff unavailable owing to an unexpected event before data collection). The third was failure by the medical secretariat to comply with the instructions in the brochure.

**Table 2:** Feasibility problems encountered

**Feasibility problem**	**Rate (%)**	
	***Before data collection***	***After data collection***
Low implication/motivation of institution	13.5	8.3
Low hands-on implication/motivation	13.5	16.0
Low implication/motivation due to external events unrelated to the project	3.2	0.0
Staff unavailable over the phone	19.2	13.5
Staff unavailable to make an appointment	14.7	12.2
Staff did not turn up at appointment	11.5	18.6
Staff unavailable by email	15.4	15.4
Staff unavailable owing to an unexpected event	0.0	0.6
Staff misunderstood QI description in instructions brochure	41.0	44.9
Staff misunderstood QI in assessment grid	41.7	44.9
Staff misunderstood QI in assessment grid instructions	38.5	39.1
Non-compliance with instructions by archives department	8.4	
Non-compliance with instructions by department of medical information	8.3	
Non-compliance with instructions by person in charge of data collection	3.8	
Non-compliance with instructions by medical secretariat	0.0	
Non-compliance with instructions by the physician	1.0	
Non-compliance with protocol instructions for other reasons	6.4	
Difficulty for CRA to access all data in the medical records	15.4	

The overall rates of occurrence of feasibility problems for each QI were 16.1% for medical record conformity, nutritional disorders, and discharge letters, and 16.7% for pain assessment. No CRA reported a problem that was an in-built limitation on feasibility. No CRA and no person in charge of data collection in each hospital reported a critical feasibility problem. An analysis of each problem with the working group led to improvements in the wording used in the amended version of the instructions.

### QI reliability

Inter-observer reliability was satisfactory for 3 of the 10 items of the QI for medical record conformity and very satisfactory for the remaining 7 items (κ: 0.59 – 0.97). Reliability was very satisfactory for the single item which could be computed for the 3 other QIs (κ: 0.86 for pain; 0.93 for nutritional disorder, and 0.96 for discharge letter). The internal consistency of the composite score of 10 items was acceptable (Cronbach α coefficient: 0.72). There were too few inter-item correlations to be able to delete any item (only 3 coefficients were significantly above 0.50 in absolute value).

### QI discriminatory power

**Figure 1:**
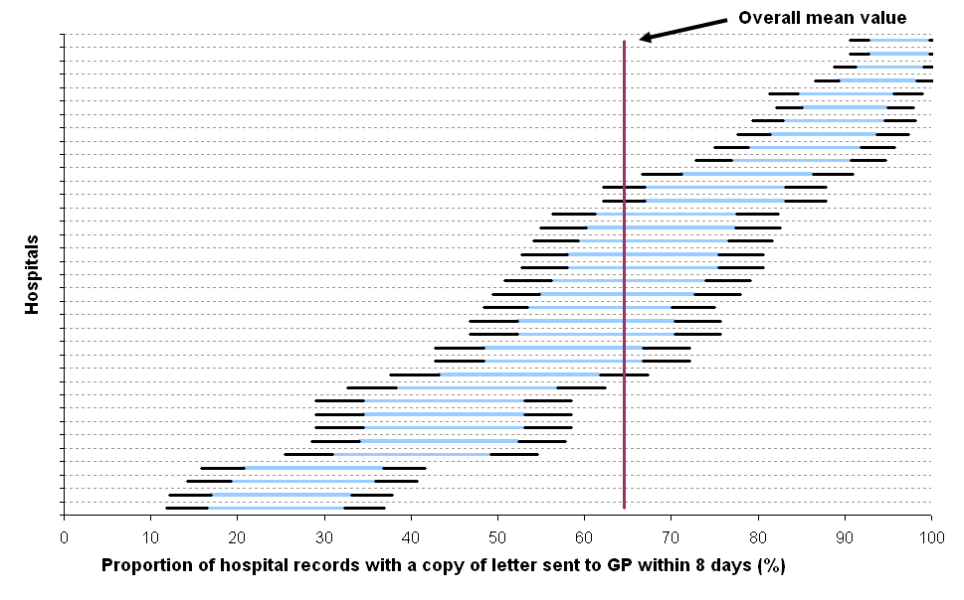
**Proportion of hospital records containing a copy of the discharge letter sent to the general practitioner within 8 days (2005).** (Light line, 99% confidence interval; dark lines, 90% confidence intervals).

The power of the QIs to discriminate among the hospitals was high as shown in Table 3. The mean ranged from 21% to 72% according to QI. In the classification of hospitals by category (see footnote to Table 3), there was at least one hospital in each category. Figure 1 illustrates the wide differences among hospitals for the QI relating to the sending of a copy of the discharge letter to the general practitioner within the legal limit of 8 days.

**Table 3:** Comparisons among hospitals based on the quality of medical records in 2005

**QI**	**Observations (N)**	**Overall mean (range)**	**Number of hospitals in each category**				
			5	4	3	2	1
Medical record conformity	2940	0.72 (0.48–0.93)	10	2	3	4	17
Traceability of pain assessment by department							
- Surgery	950	0.61 (0.03–0.98)	10	0	7	2	7
- General medicine	1171	0.21 (0–0.86)	3	3	10	1	10
- Obstetrics	462	0.34 (0–0.97)	4	1	4	0	7
- Rehabilitation	314	0.43 (0–1)	1	0	2	1	1
Screening for nutritional disorders	2312	0.70 (0.03–0.98)	12	4	9	3	8
Time elapsed before sending copy of discharge letter	2940	0.64 (0.24–0.96)	11	2	12	1	10

Category 1: both CI significantly above the mean; Category 2: 90% CI – but not 99% CI – significantly above the mean; Category 3: not significantly different from overall mean; Category 4: 90% CI – but not 99% CI – significantly below the mean; Category 5: both CI significantly below the mean.

## Discussion

We developed 4 practicable and acceptable QIs based on chart abstraction and covering different aspects of quality of care (continuity of care, staff coordination, coordination between hospitals and general practitioners, pain management and awareness of nutritional disorders). The feasibility and reliability of the 4 QIs were good. The motivation of the 36 hospitals which had volunteered to take part in the test might partly account for this result. The 4 selected QIs were also able to discriminate among hospitals, suggesting that they could be used nationwide for benchmarking purposes, to identify best performance and analyze best practice.

QI reliability was demonstrated by double-blind data collection. Since the validity of using data collected by in-house hospital staff for performing comparisons among HCOs may be questioned, external quality control of data is necessary. This is for instance the case when medical and administrative data are collected to take decisions on funding at a national level. In our study, the data was collected by external CRAs.

The burden of data collection was minimised by collecting data for all 4 QIs from a single small sample of medical records and by restricting the number of items for the QI relative to the completeness of the medical record. Sample size for 4 QIs was hardly any larger than for a single QI. Data on clinical QIs relating to specific diseases needs to be abstracted by a physician, thus increasing costs. However, our data collection did not require technical medical knowledge and could be carried out by a CRA under the supervision of a physician.

Additional improvements could further reduce the workload. Working prospectively could save the time taken to retrieve records from archives (2 days). Abstracting them directly on computer using logical and quality tests could also save time (2 days). The workload could thus be reduced from 8.5 to 4.5 days. If and when electronic medical records replace manual records [10,18], data collection and the computing of results could become fully automated.

As regards generalisation of the 4 QIs to all French hospitals, our study revealed several limitations. The first was that COMPAQH CRAs and not hospital staff assessed QI feasibility. This was so that we could assess true QI reliability and ensure good hospital participation. For hospitals to collect data there will be a need for greater transfer of knowledge and staff training. In addition, hospitals will need to find the resources to cover the cost of the 8.5 working days needed to collect data.

A second limitation concerns the items used to assess the completeness of the medical record (QI 1). These items are legal requirements and will change with changes in the law. Some legal requirements, however, are more difficult to implement than others, in particular those arising from a precautionary principle applied under public pressure. Results will then be poor and benchmarking will be impossible. An umbrella institution should thus oversee the generalization of these QIs. It could be the French National Health Directorate, which oversees quality assessment and improvement in many fields of healthcare, from general practice to HCO accreditation.

The relevance of chart abstraction itself is controversial: does the QI reflect the quality of care or just the reporting of information? If the quality improvements we have studied just boil down to improvements in reporting, this may turn out to be an inadequate response to the need to improve quality of care [19,20]. Differences in the reporting of items required by law may then have accounted for the huge differences we observed in hospital QI rates. There is, however, evidence showing that data recording does contribute to quality of care [21]. If this is indeed the case, then improving quality of care requires improving the completeness of medical records.

Finally, a sample of fewer than 100 medical records may suffice to compare hospitals, but not hospital departments, with acceptable accuracy. A department may be a more relevant unit of study than a hospital.

In conclusion, this is to our knowledge the first set of QIs using a leverage effect and lowering the burden of data collection through pooling. By using a representative panel of hospitals, trained CRAs and appropriate tools, the COMPAQH project has shown that these QIs can reveal significant differences among hospitals. The National Health Directorate has decided to include them in the next version (2009) of the accreditation procedure in which all the 3000 or so HCOs in France have to take part. Widespread use of these QIs should inspire a culture of quality measurement and the development of further QIs.

## Competing interests

The authors declare that they have no competing interests.

## Authors' contributions

EM was the principal investigator of this study and was involved in all its aspects. CC supervised data collection, was responsible for the analysis, and wrote the manuscript. VD and CG were involved in supervising data collection and participated in the analysis.

## Pre-publication history

The pre-publication history for this paper can be accessed here:


